# Pharmacological activation of FOXO3 suppresses triple-negative breast cancer *in vitro* and *in vivo*

**DOI:** 10.18632/oncotarget.9881

**Published:** 2016-06-07

**Authors:** See-Hyoung Park, Young Min Chung, Jessica Ma, Qin Yang, Jonathan S. Berek, Mickey C-T. Hu

**Affiliations:** ^1^ Division of Gynecologic Oncology, Department of Obstetrics and Gynecology, Stanford University School of Medicine, Stanford, CA 94305, USA; ^2^ Current address: Department of Biological and Chemical Engineering, Hongik University, Sejong, 339-701, Korea; ^3^ Cancer Biology Division, Department of Radiation Oncology, Washington University School of Medicine, Saint Louis, MO 63108, USA

**Keywords:** FOXO3, breast cancer, bepridil, trifluoperazine, dopamine receptor

## Abstract

Triple-negative breast cancer (TNBC) is the most lethal form of breast cancer. Lacking effective therapeutic options hinders treatment of TNBC. Here, we show that bepridil (BPD) and trifluoperazine (TFP), which are FDA-approved drugs for treatment of schizophrenia and angina respectively, inhibit Akt-pS473 phosphorylation and promote FOXO3 nuclear localization and activation in TNBC cells. BPD and TFP inhibit survival and proliferation in TNBC cells and suppress the growth of TNBC tumors, whereas silencing FOXO3 reduces the BPD- and TFP-mediated suppression of survival in TNBC cells. While BPD and TFP decrease the expression of oncogenic c-Myc, KLF5, and dopamine receptor DRD2 in TNBC cells, silencing FOXO3 diminishes BPD- and TFP-mediated repression of the expression of these proteins in TNBC cells. Since c-Myc, KLF5, and DRD2 have been suggested to increase cancer stem cell-like populations in various tumors, reducing these proteins in response to BPD and TFP suggests a novel FOXO3-dependent mechanism underlying BPD- and TFP-induced apoptosis in TNBC cells.

## INTRODUCTION

Breast cancer (BCa) is the most common cancer in women worldwide, with nearly 1.7 million new cases diagnosed and more than 520,000 related deaths in 2012 [1]. Many patients with advanced BCa will eventually have a recurrence, often with the loss of chemo-sensitivity and a poor prognosis [2–4]. Triple-negative breast cancers (TNBCs) are defined by the absence of expression of estrogen and progesterone receptors and HER2 receptors, and they account for approximately 15% of all breast cancers [5, 6]. However, TNBC has a much higher rate of mortality than non-TNBC breast cancers because treatment of this aggressive form of cancer is limited to conventional chemotherapy [7]. Currently TNBCs still have no effective targeted therapy; thus, there is clearly an unmet medical need to find new effective targeted therapeutic drugs for this disease. Although recent clinical investigations of agents such as poly(ADP-ribose) polymerase (PARP) inhibitors or EGFR inhibitors have emerged, outcomes from recent clinical trials have been rather disappointing, with substantially less clinical effectiveness detected than was expected based on preclinical and early phase trials [8–10]. It is crucial to develop novel targeted therapeutic drugs.

FOXO3 is a key protein that controls the transcription of a number of genes crucial for regulating cell cycle control [11], DNA damage and stress responses [12–15], aging and longevity [12, 16], cellular apoptosis [12, 17–19], and suppression of cancer [20–23] in animal and human cells; gene knockout findings reveal FOXO3′s additional functions in tumor suppression [24] and the maintenance of the hematopoietic stem cell pool [25]. While multiple mechanisms have been shown to regulate FOXO3 activity, phosphorylation inhibits FOXO3 nuclear translocation that controls its regulation and function. In proliferating cancer cells, survival signals trigger PI3K to activate Akt which in turn phosphorylates and inhibits specific pro-apoptotic targets such as Bad, caspase-9, and FOXO3 [12, 17]. Consequently, loss of function of FOXO3 has been linked to tumorigenesis and poor patient survival in breast cancer [20, 22, 23] and other cancers such as lung cancer [26], prostate cancer [27], and ovarian cancer [28]. In the absence of survival signal stimulation, the Akt pathway is inactivated and its cellular targets such as FOXO3 can translocate to the nucleus and induce the expression of cell cycle regulators (e.g., p27Kip1) that lead to cell cycle arrest, or upregulate certain genes (e.g., Fas-ligand and Bim) that result in apoptosis depending on the physiological conditions and the cell types [11, 12, 17–19]. The nuclear exclusion and translocation of FOXO3 into the cytoplasm inhibits FOXO3-dependent transcription. These findings suggest that FOXO3 is a pivotal tumor suppressor, and the regulation of FOXO3 activation in cancer cells may be a promising approach for developing anticancer therapeutic drugs. The identification of novel small molecules that can promote the activity of FOXO3 in TNBC cells can provide leads for new TNBC therapeutics against malignant breast tumors in which the PI3K/Akt and/or IKK/NF-kB signaling pathways are aberrantly activated, and these molecules may also result in safe and effective targeted therapy of TNBC.

In this study, we develop a new breast cancer cell-based enzymatic assay as the output to identify small-molecule drugs that increase the activity of FOXO3 by screening a druggable library of known compounds that have been approved by the Food and Drug Administration (FDA) because it has been suggested that the most fruitful basis for the discovery of a new drug is to seek new uses for old drugs [29]. Here we show anticancer properties and the preclinical therapeutic effects of two representative FDA-approved small-molecule drugs, bepridil (BPD) and trifluoperazine (TFP), which have been used clinically for treatment of schizophrenia and angina, respectively [30, 31].

Both TFP and BPD show potent cytotoxic activity in TNBC cells in culture and exhibit antitumor activity in TNBC *in vivo* models. Activation of FOXO3 tumor suppressor by TFP or BPD is a significant distinction from traditional antipsychotic inhibitors and the resultant downregulation of oncogenic survival factors, c-Myc [32] and Kruppel-like factor 5 (KLF5) [33], in TNBC is an interesting anticancer mechanism. Unexpectedly, we uncover that both TFP and BPD display suppression of the expression of the dopamine receptor D2 (DRD2), which has been suggested as a key receptor for selective-targeting cancer stem cells (CSC) [34], in a FOXO3-dependent manner. This novel finding may broaden the potential therapeutic applications beyond TNBC tumors, which are enriched with CSC characteristics.

## RESULTS

### Identification and validation of FDA-approved FOXO3-activating small-molecule drugs

**Figure 1:**
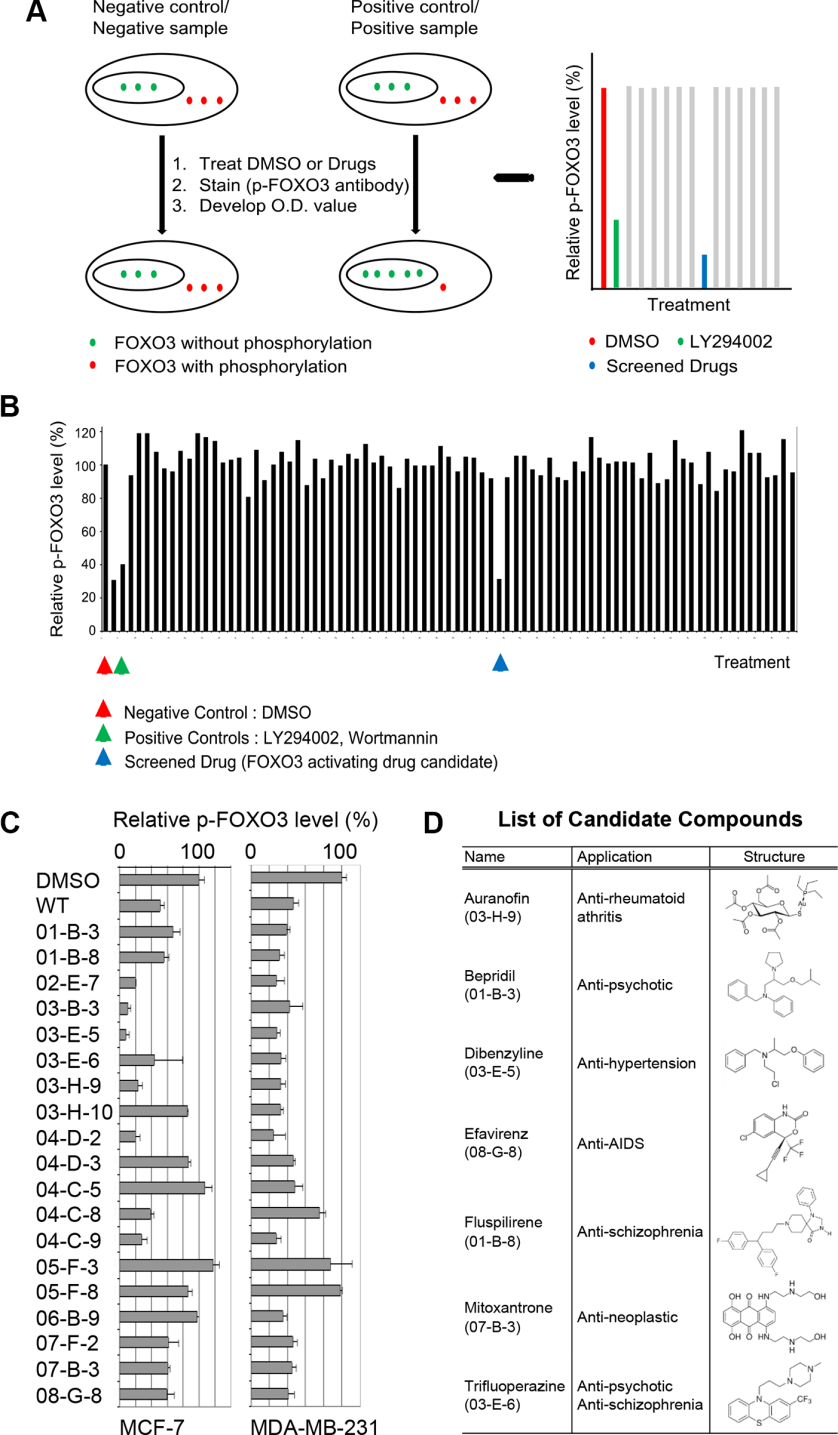
Primary and secondary screens of small-molecule drugs using a cell-based ELISA assay (**A**) Schematic diagram depicts the cell-based ELISA assay used for our drug screening. (**B**) One representative outcome of our cell-based ELISA assay is shown. In principle, breast cancer cells (e.g., MDA-MB-231) were seeded in a 96-well tissue culture plate. The cells were fixed after various treatments such as the small molecule library. After blocking, anti-phospho-FOXO3 specific antibody is incubated into the wells. The wells were washed, followed by the addition of HRP-conjugated anti-IgG secondary antibody. The wells were washed again, a substrate solution is added to the wells and color develops in proportion to the amount of protein. The Stop Solution changed the color from blue to yellow, and the intensity of the color was measured at 450 nm. (**C**) Secondary screening results obtained from MDA-MB-231 and MCF7 cells are shown. WT, Wortmanin. (**D**) The structures and original clinical applications of seven candidate compounds are shown.

To identify small molecules that can induce the activity of FOXO3 in BCa cells, we developed a new BCa cell-based enzymatic (ELISA) assay as the output to identify small molecules that can significantly inhibit the phosphorylation of Serine (S)-318/321 of FOXO3 (FOXO3-pS318/321), which is primarily localized in the cytoplasm of cells. Decreasing the level of phospho-FOXO3 leads to an increase of FOXO3 nuclear localization and its activity in BCa cells. The screening method is depicted in Figure 1A. To expedite the future clinical trials for novel lead small-molecule compounds, we screened 640 small-molecule drugs from a commercially available FDA-approved small-molecule library with this ELISA assay using a specific antibody against FOXO3-pS318/321. We used LY294002 and Wortmanin (the Akt inhibitors) as positive (inhibition) controls and DMSO as negative control. A representative screening result of our primary screen with these drugs (20 μg/ml) in MCF7 cells is shown in Figure 1B. After the primary screen, we initially selected 19 candidate small-molecule compounds for further confirmation by carrying out the secondary screen with two different BCa cell lines (MDA-MB-231 and MCF7). In total, twelve candidate compounds were confirmed, which showed a decrease of the level of FOXO3-pS318/321 around 50% in each cell line as compared with negative control (DMSO) (Figure 1C). Among them, seven top-ranked compounds were selected, which showed a decrease of the level of FOXO3-pS318/321 greater than 50% in both BCa cell lines as compared with negative control, after our secondary screens. The structures, original clinical applications, and their identification numbers corresponding to the results in Figure 1C are exhibited in Figure 1D. While these 7 drugs have no common chemical structure, two of them (BPD and TFP) have been shown to target the same protein, calmodulin, and both of them have been clinically applied to the same disorder as antipsychotic drugs [30, 31]. Thus, we focused on these two drugs for further studies.

### TFP and BPD induce nuclear localization and activating of FOXO3 in TNBC cells

To determine whether the treatment of TFP and BPD can increase the expression level of FOXO3 and its transcriptional activity, we treated TNBC MDA-MB-231 and BT549 cells with various doses of TFP or BPD for 24 hours and performed immunoblotting experiments with total lysates of these drug-treated cells. Our data show that TFP or BPD treatment leads to significant upregulation of the expression of FOXO3 and p27Kip1 and SOD2, transcriptional targets of FOXO3, in both cell lines (Supplementary Figure S1). In addition, TFP or BPD treatment significantly inhibits the phosphorylation level of Akt-Serine 473 (Akt-pS473), a key kinase that phosphorylates FOXO3, in both cell lines in a dose-dependent manner. These results suggest that TFP or BPD treatment can inhibit Akt activity, resulting in increasing FOXO3 transcriptional activity.

**Figure 2:**
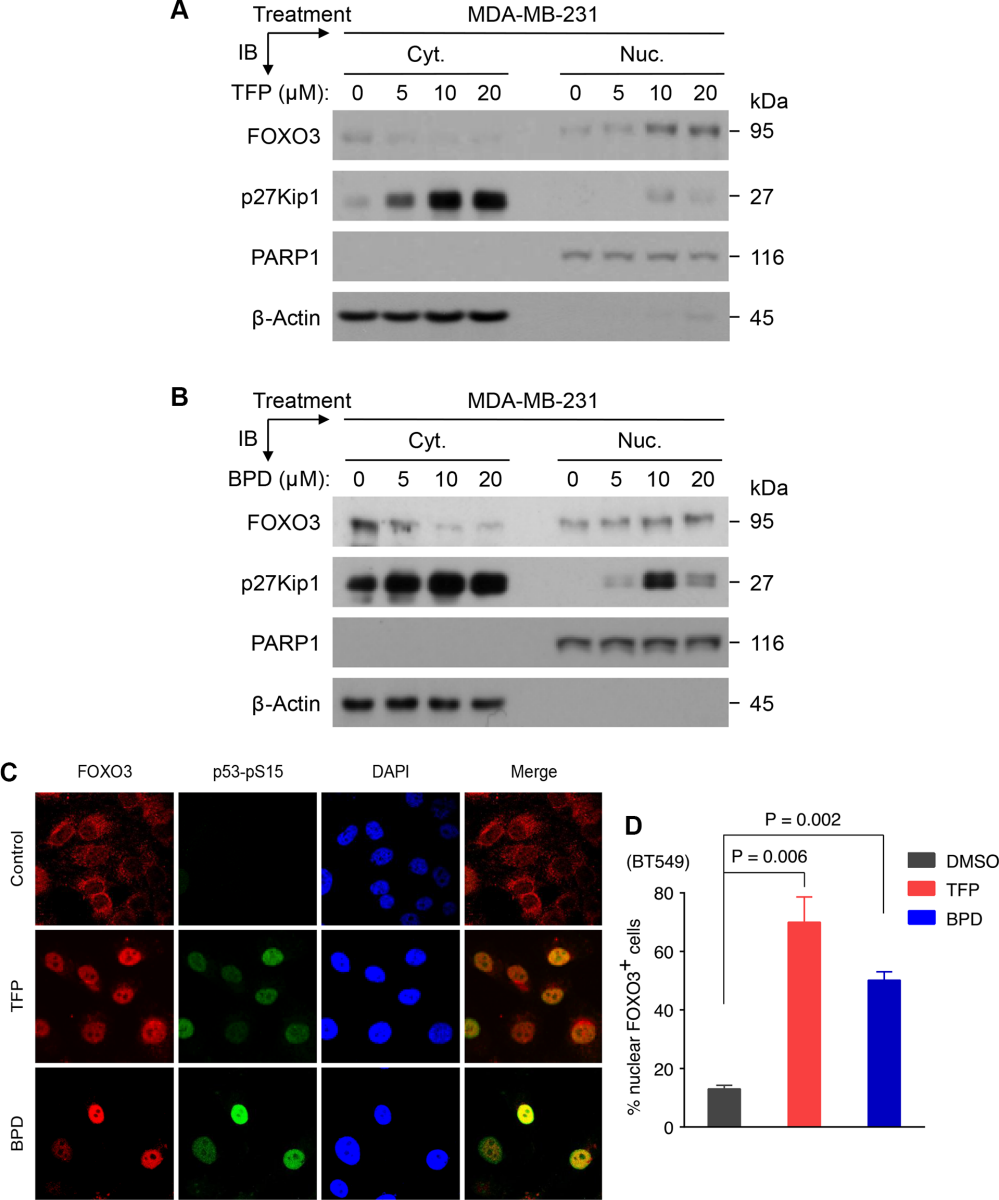
TFP or BPD treatment significantly promotes nuclear translocation and activation of endogenous FOXO3 in TNBC cells (**A**–**B**) MDA-MB-231 cells were treated with a dose response of TFP (A) or BPD (B) or dimethylsulfoxide (DMSO) control (denoted as 0 μM) for 24 hours, harvested, and fractionated for preparing cytoplasmic (Cyt.) and nuclear (Nuc.) extracts. Equal amount of each fraction was analyzed by immunoblotting (IB) analysis with specific antibodies as indicated. β-actin and PARP1 represent the fractionation and loading controls of the Cyt. and the Nuc. fractions, respectively. All these results shown above represented 3 independent experiments. (**C**–**D**) BT549 cells were treated with control (DMSO) or TFP (5 μM) or BPD (5 μM) for 24 hours. The treated cells were fixed and the subcellular localizations of endogenous FOXO3 and p53-pS15, a DNA damage marker, were detected using antibodies against FOXO3 and p53-pS15 and followed by an Alexa Fluor 594 (red)- or Alexa Fluor 488(green)-conjugated secondary antibody, and fluorescence microscopy. DAPI was used to show the nuclei, and co-localization of FOXO3 with p53-pS15 was shown as the merged images (orange or yellow color).

To determine whether TFP or BPD treatment can increase the nuclear translocation of FOXO3, we treated MDA-MB-231 and BT549 cells with various doses of TFP or BPD for 24 hours and performed immunoblotting experiments with nuclear/cytoplasmic extracts from these cells. Our data indicate that TFP or BPD treatment increases the level of nuclear FOXO3 and the expression of p27Kip1 in these cells in a dose-dependent manner (Figure 2A–2B; Supplementary Figure S2) and in a time-dependent manner (Supplementary Figures S3 and S4). Meanwhile, these results are associated with a decrease or no change of FOXO3 level in the cytoplasm of these cells. We also treated BT549 cells with TFP or BPD or control (DMSO) for 24 hours and analyzed the subcellular localization of FOXO3 as well as the phosphorylation of S-15 of p53 (p53-pS15), a hallmark of DNA damage [35], in these cells using immuno-fluorescence analysis. Our results showed that TFP or BPD promoted nuclear localization of FOXO3 (i.e., activation of FOXO3) and induced phosphorylation of p53-pS15 in BT549 cells (Figure 2C), suggesting that TFP and BPD may induce DNA damage and activate FOXO3 in TNBC cells.

### BPD and TFP suppress cell survival and promote cellular apoptosis in TNBC cells

**Figure 3:**
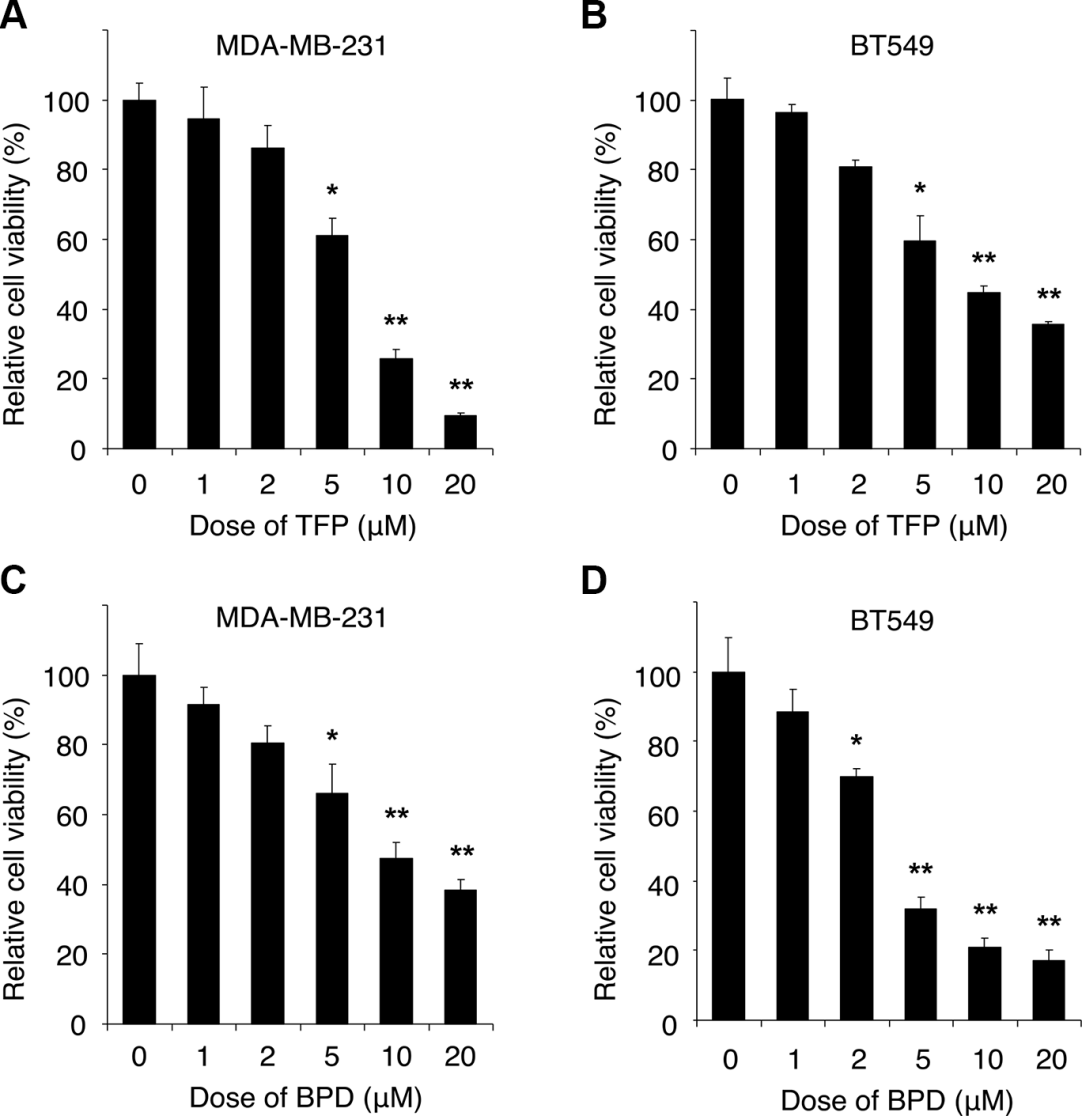
The cytotoxic effect of TFP and BPD on human TNBC cells The dose effects of TFP or BPD on cell viability of MDA-MB-231 (**A** or **C**) and BT549 (**B** or **D**) cells were determined. Cells were cultured in a 96-well plate (2,000 cells/well), treated with various doses of TFP or BPD as indicated, and incubated for 72 hours. The cell viability was determined by the MTT assay and the relative cell survival rate percentage was calculated by dividing the optical density of each drug treatment by that of the control (DMSO) treatment. Each data point represents the mean value from 3 independent experiments, with at least three replicates. The error bars represent standard deviation by paired *t*-test. The significant *P*-values between the treatment group versus the control group are indicated (*P < 0.05, **P < 0.01).

**Figure 4:**
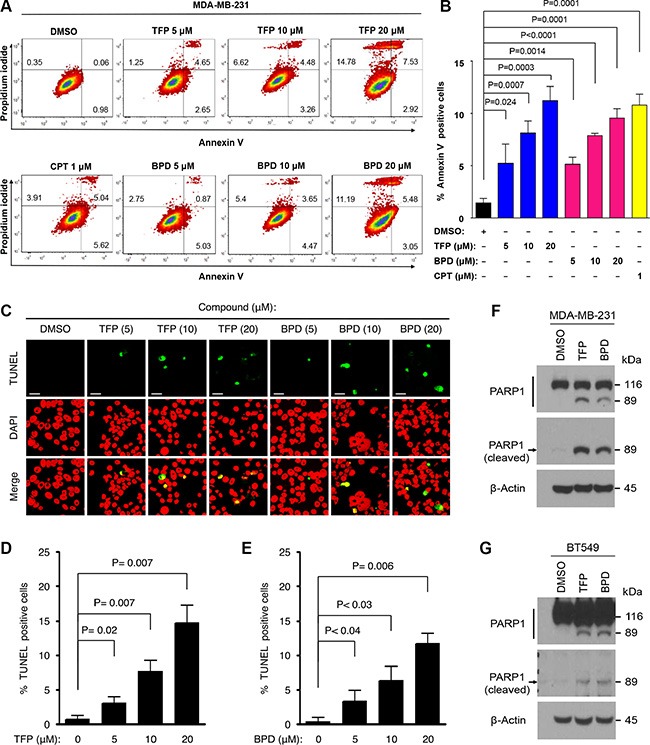
BPD and TFP promote cellular apoptosis in TNBC cells (**A**–**B**) MDA-MB-231 cells were treated with TFP or BPD or DMSO (negative control) or camptothecin (CPT) (positive control) for 48 hours, harvested, and were subjected to Annexin V and propidium iodide staining for determining apoptosis. (B) An average (%) of Annexin V-positive cells (apoptotic) cells was determined and shown in the diagram. Each data point represents the mean value from 3 independent experiments. The error bars represent standard deviation (SD). *P* values between the test-group treated with drugs versus the control-group treated with DMSO are shown. (**C**–**E**) MDA-MB-231 cells were treated with TFP or BPD or DMSO for 48 hours, and the cells were fixed on the slides for determining apoptosis by TUNEL assay. Nuclei were stained with DAPI (color-inverted to red), and merged images (yellow) were considered as apoptotic cells. Scale bar: 20 μm. (D, E) An average (%) of apoptotic (TUNEL-positive) cells was determined and shown in the diagram. The samples include three biological replicates. The error bars represent SD, and *P* values are shown. (**F**–**G**) MDA-MB-231 (F) or BT549 (G) cells were treated with TFP or BPD or DMSO for 48 hours. Total lysates of cells were analyzed by Immunoblotting analysis with specific antibodies against full-length PARP1 protein or cleaved PARP1 fragment (89 kDa) as indicated. β-actin represents the loading controls.

To determine whether BPD and TFP treatments suppress cell survival, we treated MDA-MB-231 and BT549 cells with various doses of BPD or TFP or DMSO (control) for 72 hours, and performed cell survival assays by the 3-(4,5-dimethylthiazol-2-yl)-2,5-diphenyltetrazolium bromide (MTT) assays. We found that BPD and TFP treatments significantly suppressed survival in these cells (Figure 3). To examine whether BPD and TFP treatments induce apoptosis, we treated MDA-MB-231 or BT549 cells with BPD or TFP and analyzed apoptosis by standard flow cytometry analysis with Annexin V staining, an indicator of apoptosis [36]. We showed that BPD and TFP treatments induced significant Annexin V-positive cell populations in a drug dose-dependent manner (Figure 4A–4B; Supplementary Figure S5). Moreover, we treated MDA-MB-231 or BT549 cells with various doses of TFP or BPD and analyzed apoptosis by terminal deoxynucleotidyl transferase dUTP nick end labeling (TUNEL) apoptosis assays or immunoblotting analysis of poly-ADP-ribose polymerase (PARP) degradation, an indicator of apoptosis [37], assays or DNA fragmentation assays. Our data showed that BPD and TFP treatments induced significant TUNEL-positive cells, PARP-1 degradation, and an increase in the amount of DNA fragmentation ~24 hours post drug treatments, respectively (Figure 4B–4F; Supplementary Figure S6). These results suggest that BPD and TFP may suppress cell survival and promote apoptosis in TNBC cells.

### BPD and TFP suppress the growth of TNBC tumors *in vivo*

**Figure 5:**
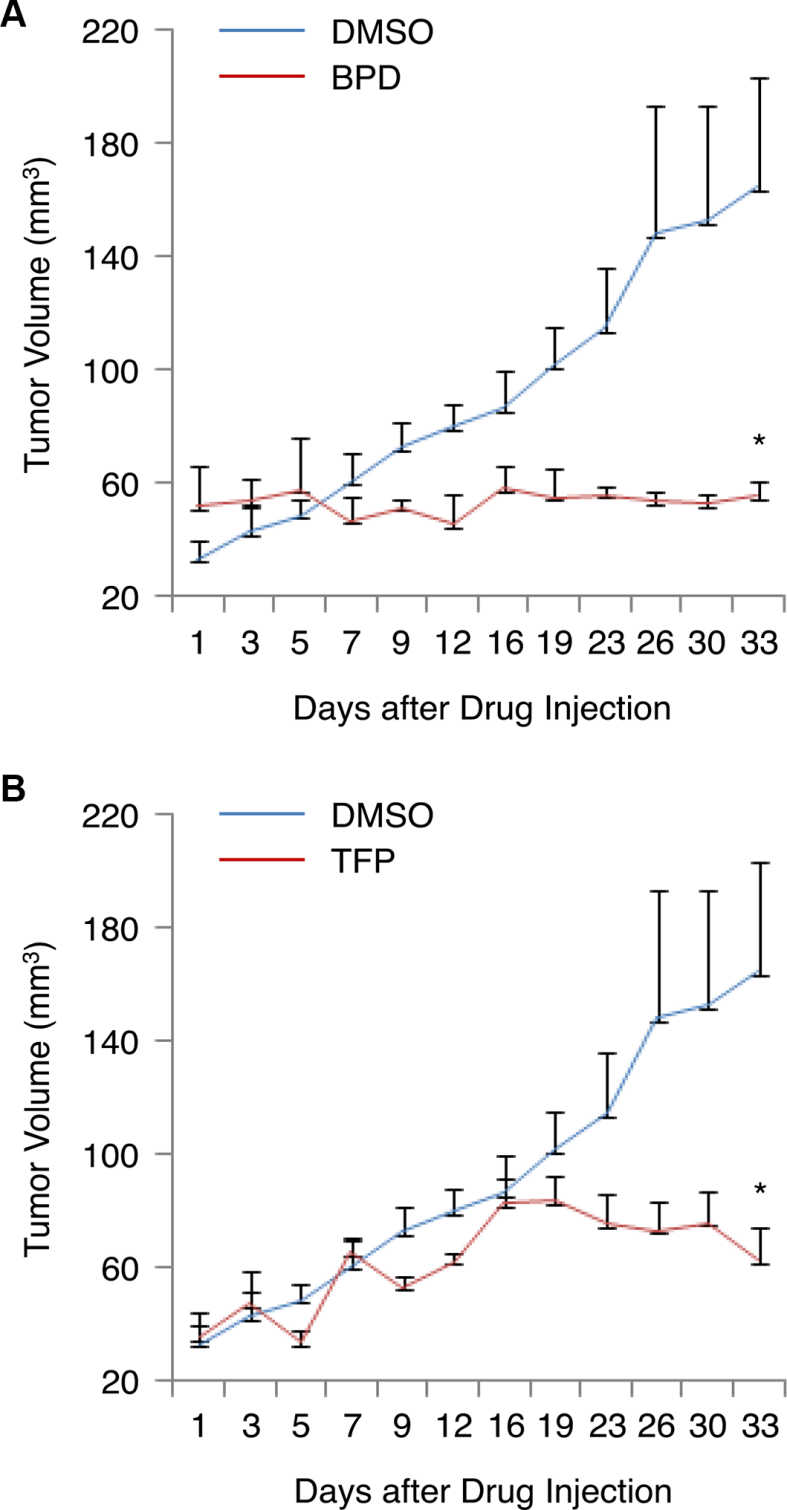
BPD and TFP suppress the growth of TNBC tumors in the mouse tumor model (**A**–**B**) MDA-MB-231 cells were injected into female nude mice (*n* = 3/group). The tumor-bearing mice were given an i.v. injection of (A) BPD (10 mg/kg) or the vehicle control (DMSO), and (B) TFP (10 mg/kg) or DMSO twice per week. The tumor volumes [(L × W^2^)/2] were measured. The results are graphed as mean ± SEM numbers of tumor volumes. Significant *P* values are shown. *P < 0.05.

To determine if treatment with BPD and TFP can suppress tumorigenesis or tumor growth in TNBC cells *in vivo*, we injected MDA-MB-231 TNBC cells into the flanks of female athymic nude mice. When palpable solid tumors were detected, the tumor-bearing mice were given an intravenous injection (0.1 ml) of BPD or TFP [10 mg/kg-body weight/mouse] or the vehicle (DMSO, negative control) three times per week at even intervals for 5 weeks. The tumor volumes were measured twice per week after the treatment. Our results show that BPD or TFP significantly suppressed MDA-MB-231 TNBC tumor growth in the mouse model (Figure 5).

### Silencing FOXO3 potently decreases the TFP- or BPD-mediated suppression of cell survival in TNBC cells

**Figure 6:**
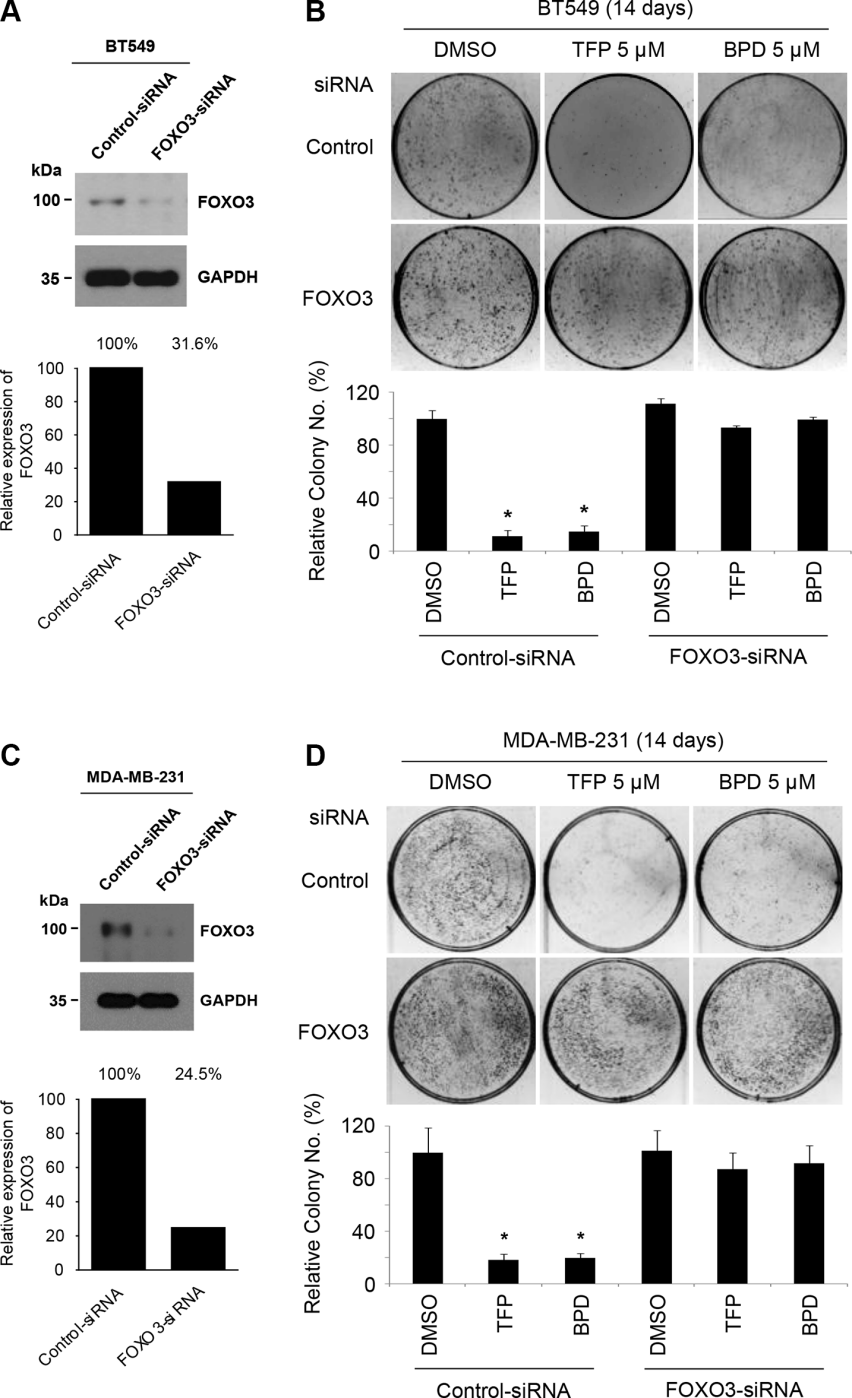
TFP and BPD suppress the colony-forming ability of TNBC cells in a FOXO3-dependent manner (**A**–**B**) (A) BT549 cells were transfected with control-siRNA or FOXO3-siRNA for 48 h. The indicated proteins were detected by immunoblotting with specific antibodies (Abs) against FOXO3 and GAPDH (loading control). (B) The transfected cells were treated with TFP, BPD or DMSO (negative control) for 14 days and stained with crystal violet solution. Top (upper panels): the representative pictures of the assays are shown. Bottom (lower panels): the numbers of colonies in the drug (TFP or BPD)-treated plates were compared with those of the DMSO-treated plates. The results are graphed as mean ± SEM numbers of cell colonies. The number of biological replicates is three. *P < 0.001 (drug vs DMSO). (**C**–**D**) (C) MDA-MB-231 cells were transfected with control-siRNA or FOXO3-siRNA, and the indicated proteins were determined as described above. (D) The transfected cells were treated with TFP, BPD or DMSO (negative control) for 14 days, stained, and the data are presented as described above.

Since it is known that FOXO3 can regulate cell survival and proliferation (cycle control) [11] and that activation of FOXO3 can promote cellular apoptosis [12, 17–19], we sought to determine whether FOXO3 is necessary for regulating TFP- or BPD-mediated inhibition of cell survival and proliferation in TNBC cells. We silenced endogenous FOXO3 in BT549 and MDA-MB-231 cells by transfecting these cells with FOXO3-siRNA or Control-siRNA (Figure 6A, 6C). Then, we treated these FOXO3-knockdown cells with TFP or BPD or DMSO (negative control) to compare the effects of these drugs on the rate of cell survival and proliferation by using the colony formation assay. While TFP or BPD treatment significantly suppressed the colony-forming ability of BT549 and MDA-MB-231 cells, silencing FOXO3 potently diminished the TFP- or BPD-mediated suppression of cell survival in these cells (Figure 6B, 6D). These results suggest that FOXO3 is essential for TFP and BPD-mediated suppression of cell survival of TNBC cells.

### TFP and BPD significantly reduce the expression of oncogenic c-Myc, KLF5, and DRD2 in TNBC cells in a FOXO3-dependent manner

**Figure 7:**
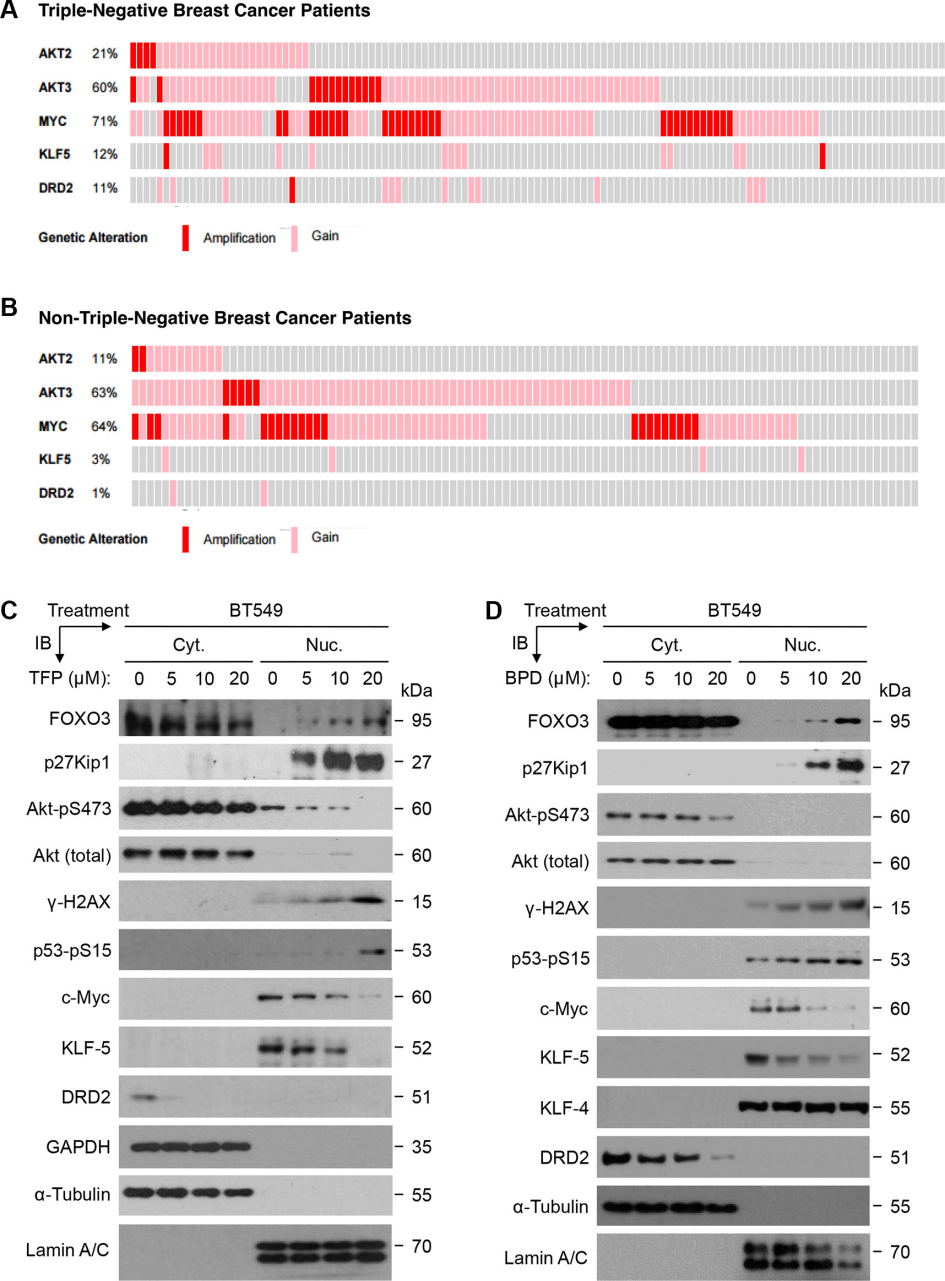
The aberrant expression of oncogenic survival proteins in TNBC tumors and the effect of TFP or BPD on these proteins in TNBC cells (**A**–**B**) Oncoprint analysis data of Akt2, Akt3, c-Myc (MYC), Kruppel-like factor 5 (KLF5), and dopamine receptor D2 (DRD2) in TNBC patient samples are displayed. Data from the TCGA dataset containing molecular subtyping data of 123 TNBC subtype (A) and 104 non-TNBC subtype (B) were extracted and used to generate an oncoprint plot using the cBioPortal [39, 40]. Gene amplification (red bars) and mRNA copy number gain (pink bars) are shown. (**C**–**D**) BT549 cells were treated with a dose response of TFP (C) or BPD (D) or DMSO control (denoted as 0 μM) for 24 hours, harvested, and fractionated for preparing cytoplasmic (Cyt.) and nuclear (Nuc.) extracts. Equal amount of each fraction was analyzed by immunoblotting (IB) analysis with specific antibodies as indicated. While α-Tubulin and GAPDH represent the loading controls of the Cyt. extract, Lamin A/C displays the loading control of the Nuc. Extract.

An important question in determining the mechanism for suppressing cell survival is how TFP and BPD contribute to the observed suppression of cell survival or growth in TNBC cells. Because it has been suggested that several cancer-promoting genes, such as Akt [38], c-Myc [32], KLF5 [33], and DRD2 [34] are involved in cell survival or proliferation in BCa cells, we sought to compare the status of these genes in human TNBC tumors versus non-TNBC tumors. Thus, we analyzed the gene amplification and mRNA copy number gain (amplification/gain) data available from The Cancer Genome Atlas (TCGA) datasets using cBioPortal [39, 40]. As shown in Figure 7A and 7B, the *Akt2* gene's amplification/gain alteration in 123 TNBC samples (21%) is about 2-fold higher than that in 104 non-TNBC samples (11%). The *Akt3 and MYC* genes' amplification/ gain alterations are high (60%–71%) in both TNBC and non-TNBC samples, while the *Akt1* gene's amplification/gain alteration is low (6% and 9% in TNBC and non-TNBC samples, respectively) (not shown). Notably, while the KLF5 gene's amplification/gain alteration in TNBC samples (12%) is 4-fold higher than that in non-TNBC samples (3%), the DRD2 gene's amplification/gain alteration in TNBC samples (11%) is 11-fold higher than that in non-TNBC samples (1%).

To confirm that TFP or BPD treatment leads to significant downregulation of the expression of key oncogenic proteins in TNBC cells, we performed immunoblotting experiments with nuclear/cytoplasmic extracts from the drug-treated cells as described above. Our data demonstrate that TFP or BPD treatment significantly suppressed of the expression of oncogenic c-Myc, KLF5, and DRD2 in BT549 cells (Figure 7C–7D). As controls, our data also show that TFP or BPD treatment potently increased the levels of FOXO3, p27Kip1, γ-H2AX, and p53-pS15 in BT549 cells, while TFP or BPD treatment decreased the phosphorylation level of Akt-pS473 as described above.

**Figure 8:**
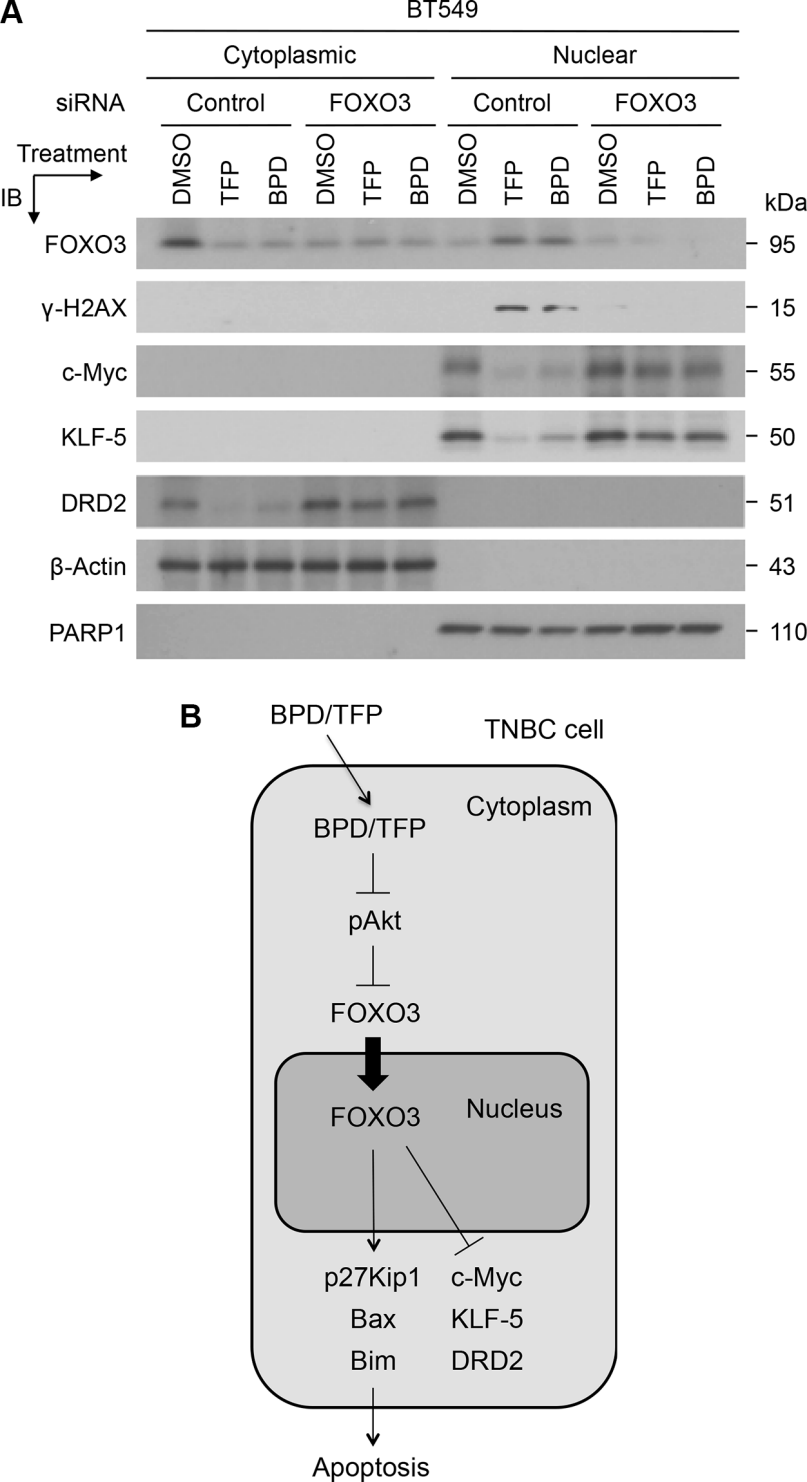
TFP and BPD significantly reduce the expression of oncogenic c-Myc, KLF5, and DRD2 in TNBC cells in a FOXO3-dependent manner (**A**) BT549 cells were transfected with control-siRNA or FOXO3-siRNA for 24 hours, transfected cells were treated with TFP, BPD or DMSO (control) for 48 hours, harvested, and fractionated for preparing cytoplasmic and nuclear extracts. Equal amount of each fraction was analyzed by immunoblotting (IB) analysis with the specific antibodies as indicated. Proteins β-Actin and PARP1 represent the fractionation and loading controls of the cytosolic and nuclear fractions, respectively. (**B**) A diagram represents the model for the FOXO3-dependent anticancer function of BPD and TFP. A schematic shows that BPD and TFP inhibit phosphor-Akt (pAkt) and lead to FOXO3 translocation from the cytoplasm into the nucleus, where FOXO3 can upregulate the expression of p27Kip1, Bax, and Bim proapoptotic proteins [17, 19, 41]. Moreover, BPD and TFP downregulate of the expression of c-Myc, KLF5 and DRD2 oncogenic survival proteins in a FOXO3-dependent manner. As a result of this FOXO3-mediated apoptotic pathway, BPD and TFP promote TNBC cell apoptosis.

To confirm that FOXO3 is essential for regulating the drug-mediated suppression of the expression of these oncogenic proteins, we transfected BT549 cells with FOXO3-siRNA or Control-siRNA (negative control) and performed immunoblotting analysis with nuclear/cytoplasmic extracts from the drug-treated cells as described above. Our results show that the expression of c-Myc, KLF5, and DRD2 in BT549-Control-siRNA cells was downregulated significantly after TFP or BPD treatment, whereas the drug-mediated downregulation of the expression of c-Myc, KLF5, and DRD2 was diminished in BT549-FOXO3-siRNA cells (Figure 8A). Similarly, the drug-mediated increase of the levels of FOXO3 and γ-H2AX was diminished in BT549-FOXO3-siRNA cells, while the levels of FOXO3 and γ-H2AX in BT549-Control-siRNA cells were increased significantly after the drug treatment (Figure 8A). Taken together, these results suggest that FOXO3 may be essential for TFP- or BPD-mediated repression of the expression of c-Myc, KLF5, and DRD2 in TNBC cells.

## DISCUSSION

Here, we provide evidence that pharmacological activation of FOXO3 by small-molecule drugs (TFP or BPD) potently suppresses cell survival and proliferation in TNBC cells *in vitro* and in TNBC tumors *in vivo*. TNBC has been clinically proven to be associated with a shorter median time to relapse and death and does not respond to endocrine therapy or other available targeted agents at present. Increased aggressiveness of this tumor, as well as resistance to standard drug therapies, may be linked to the presence of CSC populations within TNBC tumors. However, if anticancer therapies can eliminate differentiated cancer cells but cannot overcome CSC populations in TNBC tumors, a small population of tumor-initiating CSC may remain untouched and cause relapse in the future. Thus, it is important to identify new therapeutic drugs that can specifically target CSC populations in tumors. Our results show that both TFP and BPD suppress the expression of oncogenic c-Myc, KLF5, and DRD2 proteins, and silencing FOXO3 significantly diminishes TFP- or BPD-mediated repression of the expression of these proteins in TNBC cells. In addition to upregulation of p27Kip1, activation of FOXO3 upregulates pro-apoptotic Bax and Bim proteins [17, 19, 41]. Thus, we have proposed a schematic representation of the FOXO3-dependent promotion of apoptosis in TNBC cells in response to the treatment of BPD or TFP (Figure 8B).

It has been shown that Akt is aberrantly expressed or constitutively activated in several cancers including BCa [38]. Based on the relative phosphorylation status of FOXO3 (Ser318/321) by Akt in cells treated with drugs, we have developed a new cell-based screening system for selecting small-molecule drugs that promote FOXO3 nuclear localization in BCa cells. Through this novel screening system, we have identified and validated several candidate drugs from an FDA-approved small-molecule library. A key advantage of repurposing FDA-approved drugs is that the toxicity profiles, pharmacokinetic, and pharmacodynamic studies of these drugs have already been approved by the FDA; thus, saving cost and time for new drug development [29]. In addition, based on the known activities of existing drugs, the mechanisms of new actions of these drugs tend to be more predictable and successful than those made of entirely unknown compounds. For examples, while aspirin has been previously used as an analgesic to relieve pain, it has been recently shown to exert anti-platelet activity and is rapidly being repurposed for preventing cardiovascular diseases [42]. Before thalidomide was found to cause birth defects, it was used to treat morning sickness in pregnant women. When it was found to cause birth defects, it was taken off the market. Later, through drug repurposing, it became available again as a treatment for multiple myeloma and leprosy. Milnacipran was initially used as an antidepressant drug but it became one of three modern drugs for treating fibromyalgia. Finally, thioridazine, an anti-psychotic drug, was repurposed to selectively inhibit the development of acute myeloid leukemia derived from their CSC through antagonizing DRD2 [34].

We focused on two drugs (TFP and BPD) based on their same target and similar clinical applications. In agreement with our findings, TFP has been shown to induce apoptosis in A549 lung cancer cell line that is associated with downregulation of anti-apoptotic Bcl-2 protein and upregulation of pro-apoptotic Bax protein [43]. BPD has recently been shown to stimulate the tumor necrosis factor-related apoptosis-inducing ligand (TRAIL)-mediated apoptosis in U251MG glioblastoma cell line via upregulation of the expression of death receptor 5 (DR5) [44]. Interestingly, FOXO3 can upregulate the expression of TRAIL in prostate cancer cells [45], and FOXO3 may be essential for the expression of DR5 in colon and live cancer cells [46, 47]. However, the roles and mechanisms of TFP and BPD in suppressing TNBC cell survival and tumor development are unknown and have not yet been explored. TFP and BPD are known to be calmodulin antagonists, which have been used clinically for treating psychotic disorders such as schizophrenia and cardiac arrhythmia. Our current data indicate a successful case of repurposing TFP and BPD for treating TNBC. More importantly, we show that TFP and BPD treatments promote suppression of the expression of specific oncogenes (c-Myc, KLF5, and DRD2), which are suggested to increase CSC-like populations in various tumors [34, 48–50]. Also, our data suggest an essential role of FOXO3 in TFP/BPD-induced downregulation of these CSC-related oncogenic proteins, which is consistent with our recent finding suggesting that FOXO3 may be necessary for certain drug-mediated suppression of the expression of markers (such as c-Myc and CD44) exhibiting CSC characteristics in TNBC cells [51]. Moreover, TFP has a very similar chemical structure to thioridazine, a CSC targeting drug that selectively inhibits CSC [36], and both drugs exert similar pharmacological effects when clinically treating psychotic disorders. Collectively, our novel findings suggest that TFP and BPD may become potential therapeutic drugs for TNBC, although further investigation of this therapeutic concept of targeting CSC in TNBC tumors with TFP and BPD (or their analogs) is necessary to establish novel therapeutic interventions in order to conquer malignant TNBC in the future.

## MATERIALS AND METHODS

### Cell culture, cell lines, shRNA and siRNA transfection

BT549 (human breast epithelial ductal carcinoma), MDA-MB-231 (human breast invasive ductal carcinoma), and MCF7 (human breast adenocarcinoma) cell lines were obtained from the American Type Culture Collection, and grown in DMEM/F12 supplemented with L-glutamine (3%), penicilline/streptomycin (1%) and 10% fetal bovine serum at 37°C in a humidified CO_2_ incubator (5% CO_2_). Specific siRNA against FOXO3 (sc-37887) and control-siRNA (sc-44231) were obtained from Santa Cruz Biotechnology (SCBT) (Santa Cruz, CA). For transfection with siRNA, cells were transfected with specific siRNA or control siRNA as indicated by using DharmaFECT 1 transfection reagent (Thermo Scientific, Rockford, IL) according to the manufacturer's protocol.

### Antibodies and reagents

TFP, dimethylsulfoxide (DMSO), 3-(4,5- dimethylthiazol-2-yl)-2,5-diphenyltetrazolium bromide (MTT), and 3,3′,5,5′-Tetramethylbenzidine (TMB) were purchased from Sigma (St. Louis, MO). Propidium iodide (PI) was obtained from Fisher Scientific. BDP and antibodies specific to FOXO3 (FKHRL1) N-16 (1:500 dilution) and H-144 (1:1000 dilution), p53-pS15 (1:1000 dilution), c-Myc (1:1000 dilution), α-Tubulin (1:2000 dilution), PARP1 (1:1000 dilution), SOD2 (1:2000 dilution), and Lamin A/C (1:1000 dilution) were purchased from SCBT. Antibody against phospho-H2AX Serine-139 (1:1000 dilution) was obtained from Millipore (Billerica, MA). Specific antibodies against phospho-FOXO3-S318/321 (#9465, 1:250 dilution), Akt-pS473 (1:1000 dilution), Akt (total, 1:1000 dilution), and p27Kip1 (1:1000 dilution) were purchased from Cell Signaling Technology (Danvers, MA). Antibodies against FOXO3 (2071-1 and 3280-1) (1:1000 dilution) were obtained from Epitomics (Burlingame, CA). Antibody against β-Actin (1:3000 dilution) was purchased from Sigma. Antibody against GAPDH (1:1000 dilution), Alexa 488- and Alexa 594-conjugated secondary antibodies were obtained from Thermo-Fisher Scientific (Waltham, MA). Goat anti-mouse IgG and goat anti-rabbit IgG horseradish peroxidase-conjugated secondary antibodies were purchased from Jackson ImmunoResearch (West Grove, PA).

### Cell-based ELISA assay

Cells (2 × 10^4^/well in 100 μl medium) were seeded into 96-well black microplates and incubated at 37°C for 24 hours in a CO_2_ incubator. Cells in each well were treated separately with DMSO (negative control), LY294002 or Wortmannin (negative controls), and small-molecule compounds (20 μg/ml final conc.) from the FDA-Approved Drug Library (from Enzo Life Sciences International, Inc) (totally 640 drugs, 80 drugs per 96-well plate) at 37^°^C and 5% CO_2_ for 24 hours. Then, cells were fixed and quenched by adding 100 μl of 4% formaldehyde [in phosphate-buffered saline (PBS)] and 100 μl of 0.6% H_2_O_2_ (in PBS), respectively. To determine the level of the phosphorylated-FOXO3-S318/321 in these cells, ELISA was performed by treating cells with 100 μl of the blocking buffer [5% bovine serum albumin (BSA) in Tris-buffered saline, 0.1% Tween-20 (TBST)] for I hour. After washing, cells were treated with 100 μl of primary antibody against phospho-FOXO3-S318/321 (1:250 dilution in TBST containing 2% BSA) at 4^°^C for overnight. After washing, cells were treated with 100 μl of the anti-rabbit IgG horseradish peroxidase-conjugated secondary antibody (1:1000 dilution in TBST containing 2% BSA) at room temperature for 2 hours. After washing, cells were treated with 100 μl of peroxidase substrate (TMB) for 15 minutes, followed by adding 50 μl of ELISA stop solution (2N H_2_SO_4_). The optical density of each well was measured by reading the microplate using a microplate reader at 450 nm.

### Immunofluorescence analysis

Experiments were essentially performed as described [41, 51]. Briefly, BT549 cells were treated with TFP (5 μM) or BPD (5 μM) or vehicle (DMSO) for 24 hours, fixed, and permeabilized. After blocking with BSA, cells were incubated with a primary antibody against FOXO3 (1:50 dilution) or p53-pS15 (1:100 dilution), followed by Alexa 594 (red)-conjugated anti-rabbit (1:200 dilution) or Alexa 488 (green)-conjugated anti-mouse (1:200 dilution) secondary antibody, respectively. After counterstaining with DAPI, fluorescence images were captured with a confocal microscope. The percentage of nuclear FOXO3-positive cells was quantitated by analyzing ~100 cells images randomly captured by confocal microscopy with the Image J software (v.1.49).

### Cytoplasmic and nuclear protein fractionation

Experiments were performed as described [19, 41]. Briefly, TNBC cells from various treatment conditions were trypsinized, centrifuged, washed, re-suspended in a cytoplasmic fractional buffer and incubated at 4°C for 30 min. The cell suspension was centrifuged at 10,000 rpm for 30 min at 4°C and the supernatant was collected for cytoplasmic fraction. The nuclear pellet was washed twice with the washing buffer. The remaining pellet was re-suspended with a nuclear fractional buffer and incubated at 4°C for 30 min. The nuclear suspension was centrifuged at 13,000 rpm for 30 min at 4°C, the supernatant was collected for nuclear fraction. Protein in each fraction was quantified as described [19, 41].

### Immunoblotting analysis

For immunoblotting (Western blotting) analysis, equal amount (20–50 μg) of each protein sample was subjected to SDS-PAGE (8%–12%) and transferred onto nitrocellulose membranes as described [19, 41, 51]. Briefly, membranes were blocked in 3% BSA in TBST buffer and incubated for 1 hour with a primary antibody. After three washes with TBST, membranes were incubated for 1 hour with horseradish peroxidase-conjugated secondary antibodies (1:3000 or 1:5000 dilution). The immunoblots were visualized by enhanced chemiluminescence as described [19, 41, 51].

### MTT assays

TNBC cells (1 × 10^3^/well) were seeded into 96-well plates and incubated at 37°C in a CO_2_ incubator (5% CO_2_) overnight. TNBC cells were treated with DMSO (control) and various doses of TFP or BPD as indicated for 72 hours, followed by the addition of 20 μl of MTT solution (5 mg/ml in phosphate buffer). After incubation for 2 hours, the solution in each well was removed, and then the blue crystalline precipitate in each well was dissolved in DMSO (200 μl/well). The visible absorbance at 545 nm of each well was quantified using a microplate reader.

### Annexin V staining fluorescence-activated cell sorting (FACS) analysis

The percentage of cells that are undergoing apoptosis was determined by using the FITC Annexin V Apoptosis Detection Kit I (BD PharMingen) with propiodium iodide according to the manufacturer's instructions. After treatment with various doses of TFP or BPD or DMSO (negative control) or camptothecin (positive control) for 48 hours, cells were washed in PBS, trypsinized, and resuspended in binding buffer [10 mM Hepes/NaOH (pH 7.4), 140 mM NaCl, 2.5 mM CaCl2] at a density of 1 × 10^6^ cells/ml.

Afterwards, the cells were aliquoted into 5 ml culture tubes (1 × 10^5^ cells/tube) and incubated in binding buffer containing 5 μl of FITC Annexin V and 5 μl of PI for 15 min at 25°C in the dark. Cells were analyzed by using a FACScan or FACSCalibur (BD Biosciences) at the Institutional shared FACS Facility and the data were analyzed by FlowJo or FCS 5 Express (De Novo Software, CA). Ten thousand events were collected in each run. Each data point represents the mean value from 3 independent experiments. The error bars represent standard deviation (SD), and the statistical test is the unpaired *t*-test.

### Terminal deoxynucleotidyl transferase dUTP nick-end labeling (TUNEL)

Experiments were performed as described [41]. Briefly, MDA-MB-231 cells were grown on glass coverslips. After treatment with various doses of TFP or BPD or DMSO (control) for 48 hours, cells were fixed with 4% paraformaldehyde solution and permeabilized with Triton X-100 (0.2%). For TUNEL assay, cellular apoptosis assay was determined by enzymatic labeling of DNA strand breaks with a TUNEL assay kit (the DeadEnd Fluorometric TUNEL System, Promega) according to the manufacturer's instructions. Nuclei were stained with DAPI (color was inverted to red). Merged images (yellow) were considered as apoptotic cells and counted under a confocal laser-scanning microscope (Leica SP2 AOBS). The samples include three biological replicates, the error bars represent SD, and the statistical test is the paired *t*-test.

### DNA fragmentation assay

TNBC cells (2 × 10^7^ per sample) were trypsinized, lysed in the lysis buffer (10 mM Tris-HCl, 10 mM EDTA, 0.1% Triton-X 100, 0.1% SDS and pH 7.5) and incubated on ice for 30 min. The lysates were digested with RNase I followed by digestion with proteinase K. The DNA was extracted by phenol-chloroform (1:1, v/v), precipitated with 2 volumes of EtOH plus 10% NaAc (3 M, pH 5.2) and then dissolved in distilled water. Equal amounts of the extracted DNA (2 μg/lane) and size markers (1-kb ladder) were subjected to electrophoresis on 2% agarose gels, which were stained with ethidium bromide and photographed.

### Colony formation assay

TNBC cells (0.5 × 10^3^) were seeded into 6 cm dishes and incubated at 37°C in a humidified incubator containing 5% CO_2_ for 18 hours. After incubation, cells were treated with DMSO as control vehicle and TFP (5 μM) or BPD (5 μM) for 14 days. Then, the colonies were washed twice with PBS, fixed with 3.7% Paraformaldehyde, and stained with 1% crystal violet solution in distilled water. Experiments were performed three times.

### Animal studies

To determine the antitumor effect of TFP and BPD *in vivo*, female athymic (*nu/nu*) nude mice were purchased from Charles River Laboratories, Inc. (Wilmington, MA) and maintained aseptically in an athymic animal room. For tumor-cell implantation, MDA-MB-231 cells (in log-phase growth) were harvested, washed with PBS, and re-suspended in PBS. Then cells (5 × 10^6^ in 0.1 ml PBS with 30% ECM gel) were injected subcutaneously into the flank of each mouse as described previously [51, 52]. When palpable solid tumors were detected (approx. 100 mm^3^), each group of mice was given an *intravenous (i.v.)* injection (0.1 ml) of BPD or TFP (10 mg/kg-body weight/mouse) three times per week at even intervals for five weeks. The vehicle (DMSO) was used as a negative control group. The tumor sizes were measured twice per week with a Vernier caliper. Data are presented as means and standard deviations (SD) of four mice in each group. Mice were weighed twice per week as a measure of overall systemic toxicity. All procedures were performed in compliance with the guidelines and regulations of the Institutional Animal Care and Use Committee (IACUC). The IACUC has approved the experiments listed in the Protocol number 21557 (Stanford's Animal Welfare Assurance Number: A3213-01).

### Statistical analysis

All data are expressed as means and SD from at least three determinations. The statistical significance of difference in nuclear localization of proteins examined in cells (by immunofluorescence) and in the percentage of cell growth/survival (by MTT assay) or apoptotic nuclei (by TUNEL assay) between two groups was analyzed with two-sided unpaired Student's *t* tests when the variances were equal with Grahpad PRISM (Ver.6.07) statistical software (San Diego, CA) or Excel (Microsoft Office). All statistical tests were two-sided, and *P* values less than 0.05 were considered statistically significant.

## SUPPLEMENTARY FIGURES AND TABLES


