# Supplementary material for: Pharmacological activation of FOXO3 suppresses triple-negative breast cancer in vitro and in vivo
Source: Oncotarget. 2016 Jun 7;7(27):42110–25. doi: 10.18632/oncotarget.9881 (PMC5173120; doi:10.18632/oncotarget.9881)
Supplement: Supplementary file 1 [file oncotarget-07-42110-s001.pdf]

Pharmacological activation of FOXO3 suppresses triple-negative breast cancer *in vitro* and *in vivo*

Supplementary Materials

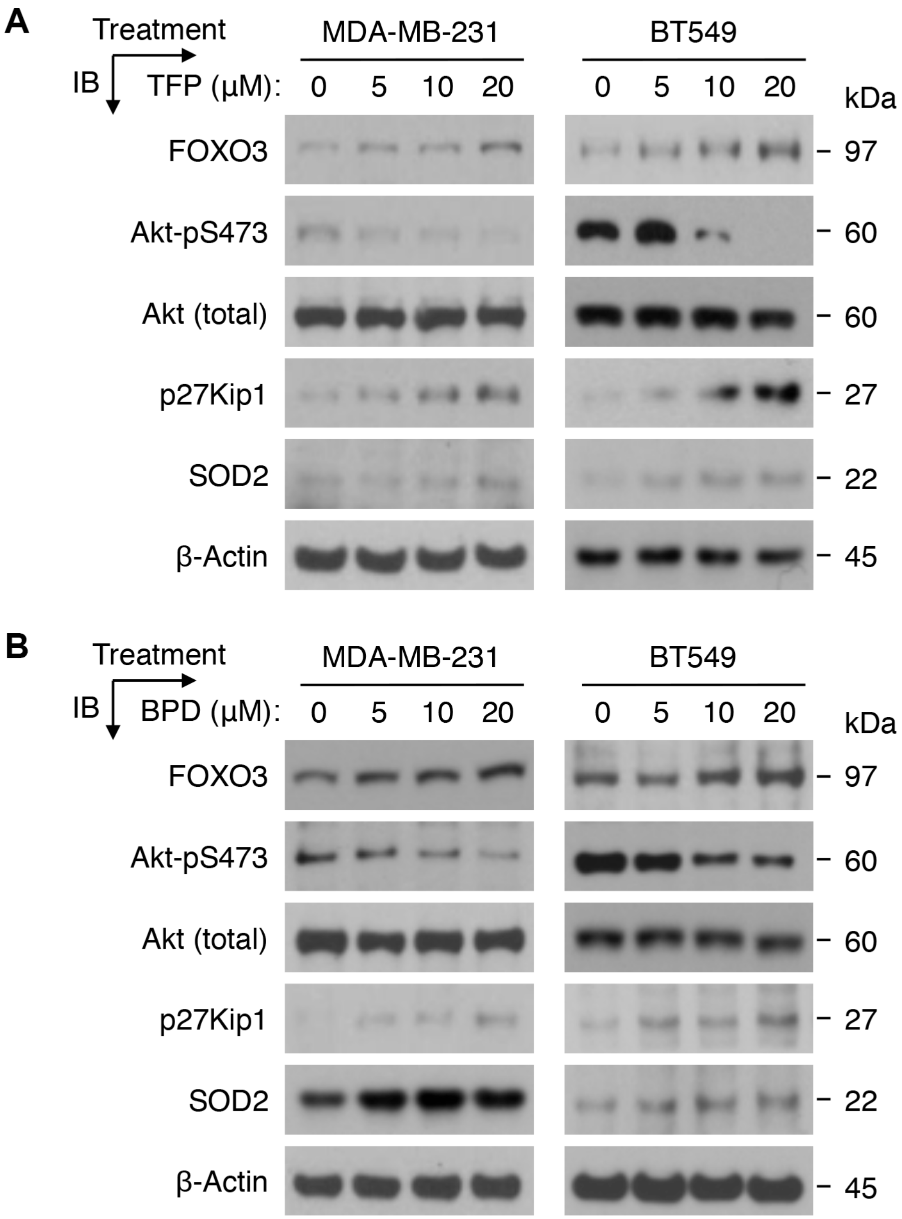

Supplementary Figure S1: While TFP and BPD induce significant upregulation of the expression of FOXO3 and p27Kip1 and SOD2 in TNBC cells, TFP and BPD markedly inhibit the phosphorylation level of Akt-pS473 in both cell lines. MDA-MB-231 and BT549 cells were treated with various doses of (**A**) TFP or (**B**) BPD or dimethylsulfoxide (DMSO) control (denoted as 0 µM) for 24 hours, and total lysates from these cells were subjected to immunoblotting (IB) analysis using specific antibodies as indicated. β-Actin represents the loading controls.

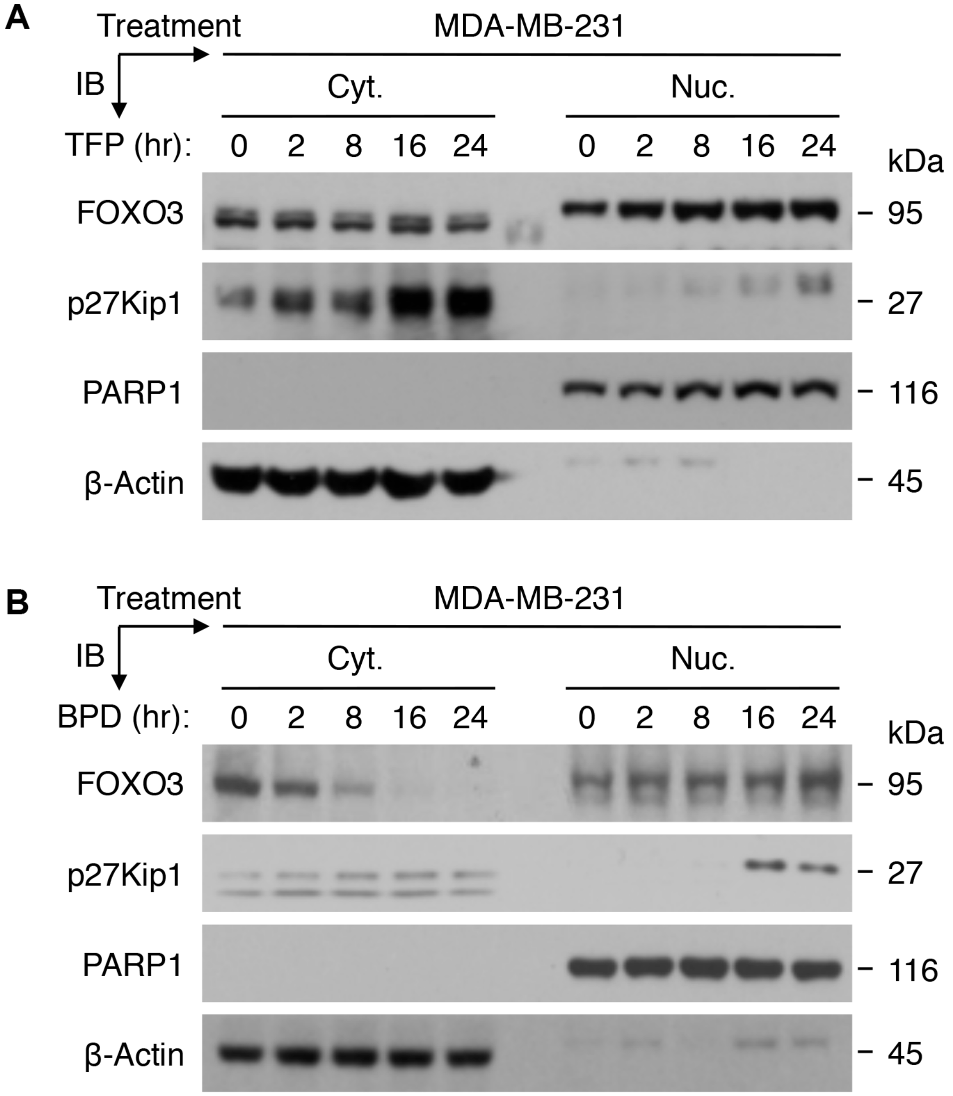

Supplementary Figure S2: TFP or BPD treatment significantly promotes nuclear translocation and activation of endogenous FOXO3 in a time-dependent manner. MDA-MB-231 cells were treated with (**A**) TFP (20 μM) or (**B**) BPD (20 μM) for a time course as indicated, harvested, and fractionated for preparing cytoplasmic (Cyt.) and nuclear (Nuc.) extracts. Equal amount of each fraction was analyzed by immunoblotting (IB) analysis with specific antibodies asindicated. β-actin and PARP1 represent the fractionation and loading controls of the Cyt. and the Nuc. fractions, respectively. All these results shown aboverepresented 3 independent experiments.

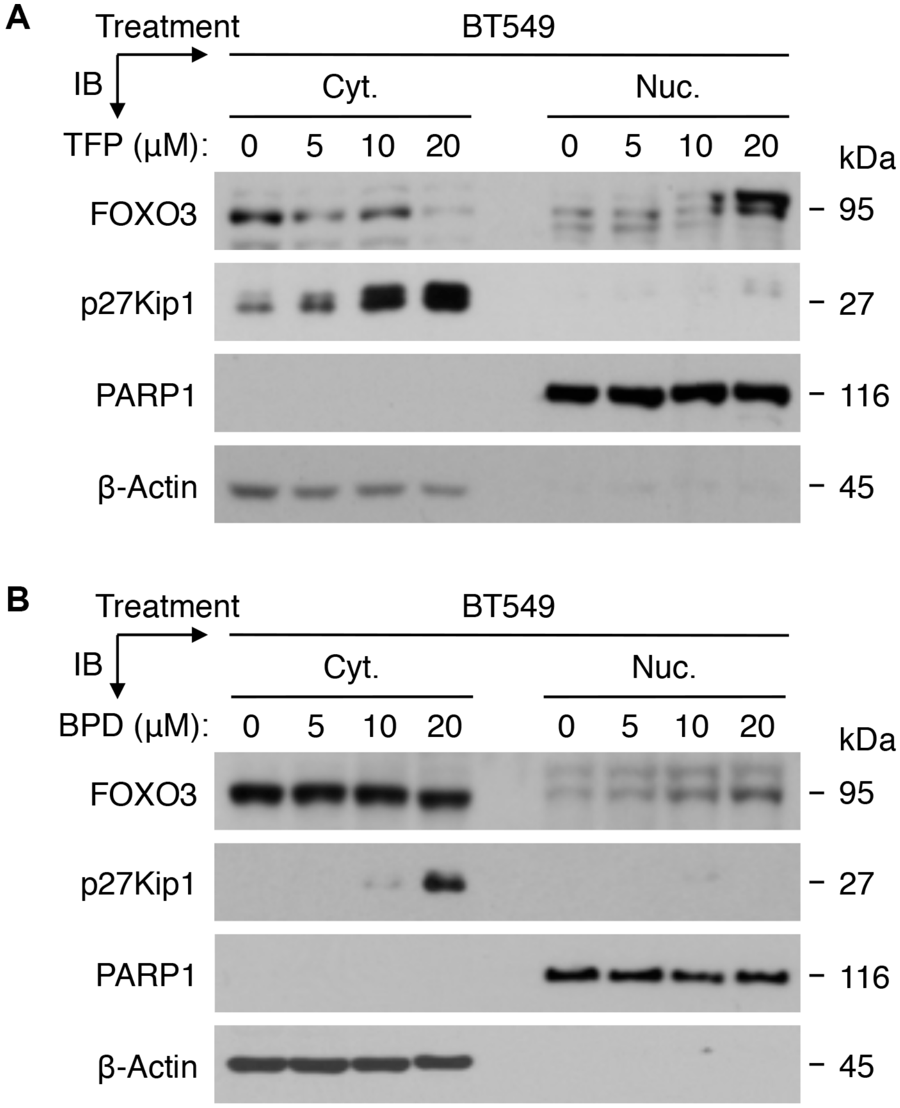

Supplementary Figure S3: TFP or BPD markedly promotes nuclear translocation and activation of endogenous FOXO3 in BT549 cells in a dose-dependent manner. BT549 cells were treated with various doses of (**A**) TFP or (**B**) BPD as indicated, harvested, and fractionated for preparing cytoplasmic (Cyt.) and nuclear (Nuc.) extracts. Equal amount of each fraction was analyzed by immunoblotting (IB) analysis with specific antibodies as indicated. β-actin and PARP1 represent the fractionation and loading controls of the Cyt. and the Nuc. fractions, respectively. All these results shown above represented 3 independent experiments.

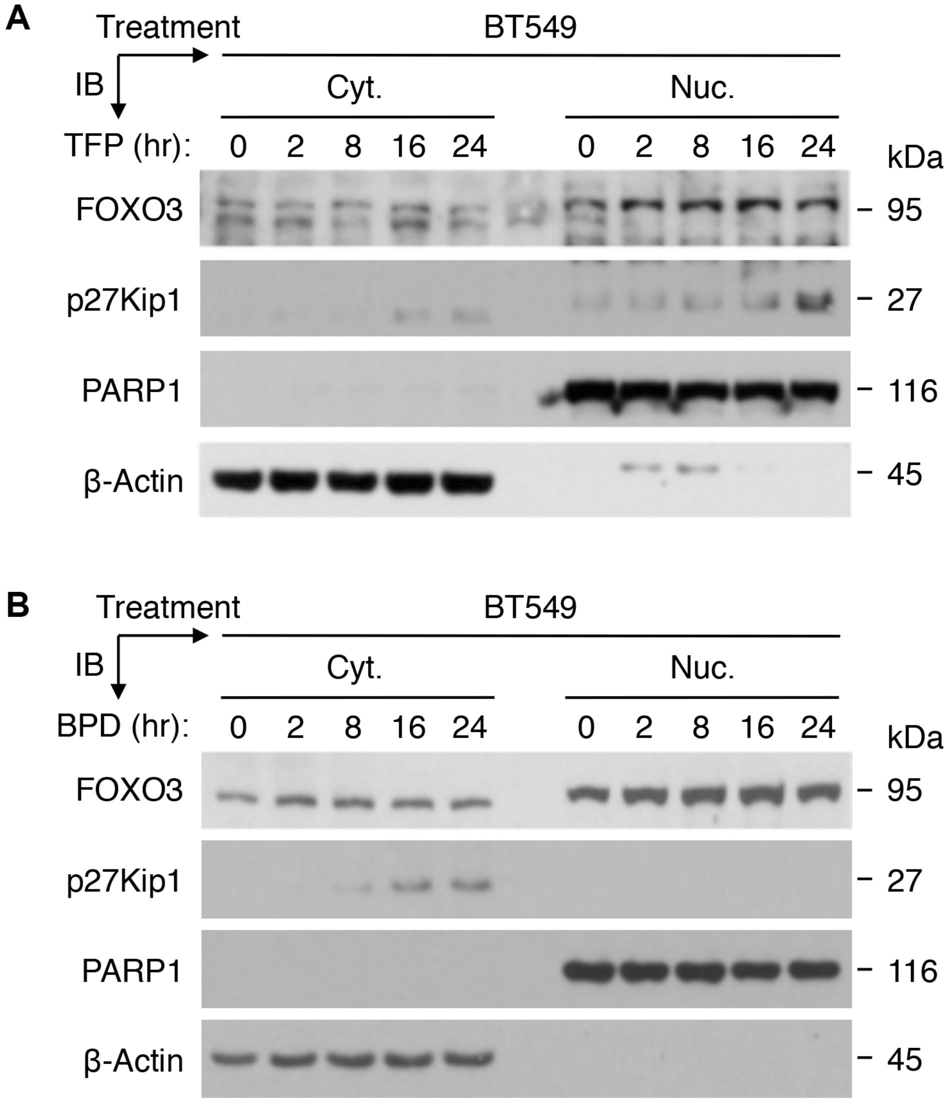

Supplementary Figure S4: TFP or BPD treatment promotes nuclear translocation and activation of endogenous FOXO3 in BT549 cells in a time-dependent manner. BT549 cells were treated with (**A**) TFP (20 μM) or (**B**) BPD (20 μM) for a time course as indicated, harvested, and fractionated for preparing cytoplasmic (Cyt.) and nuclear (Nuc.) extracts. Equal amount of each fraction was analyzed by immunoblotting (IB) analysis with specific antibodies as indicated. β-actin and PARP1 represent the fractionation and loading controls of the Cyt. and the Nuc. fractions, respectively. All these results shown above represented 3 independent experiments.

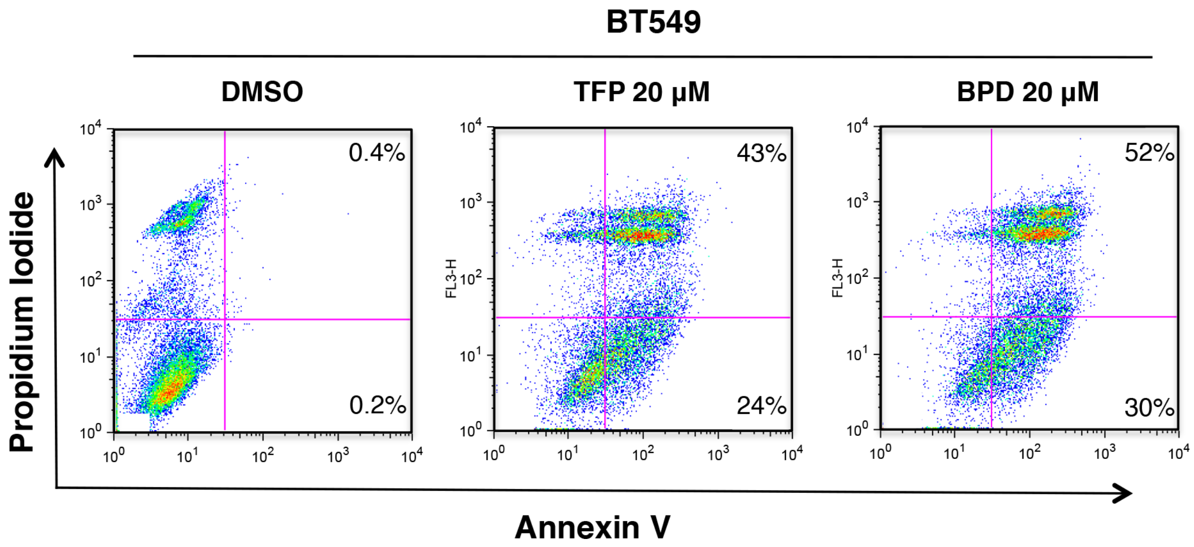

Supplementary Figure S5: TFP or BPD treatment significantly promotes cellular apoptosis in BT549 cells. BT549 cells were treated with TFP or BPD or DMSO (negative control) for 48 hours, harvested, and were subjected to Annexin V and propidium iodide staining for determining apoptosis.

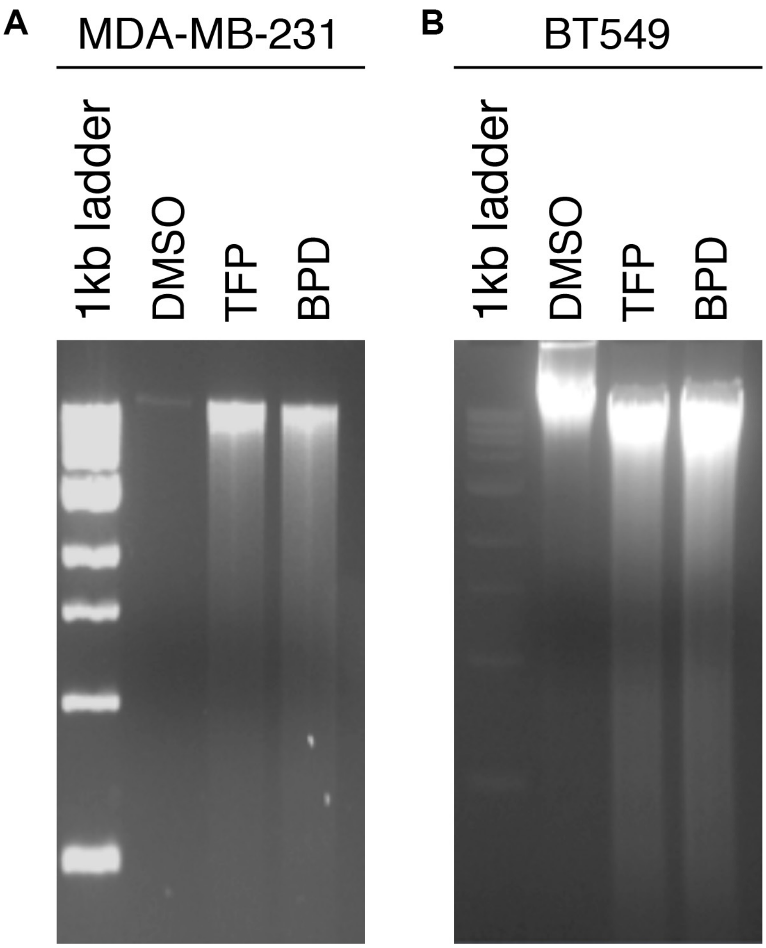

Supplementary Figure S6: TFP or BPD treatment induces cellular apoptosis in MDA-MB-231 and BT549 cells. (**A**) MDA-MB-231 and (**B**) BT549 cells were treated with TFP (20 µM) or BPD (20 µM) or DMSO for 48 hours. DNA samples extracted from these cells and subjected to DNA fragmentation assay. Equalamounts of the extracted DNA (2 µg/lane) and size markers (1-kb ladder) were subjected to electrophoresis on 2% agarose gels, which were stained with ethidium bromide and photographed.
